# *Lactobacillus helveticus* Induces Two Types of Dendritic Cell Activation and Effectively Suppresses Onset of the Common Cold: A Randomized, Double-Blind, Placebo-Controlled Trial

**DOI:** 10.3390/nu17010101

**Published:** 2024-12-30

**Authors:** Hiroka Wada, Takashi Mawatari, Yasuo Saito, Naoki Azuma, Yoshitaka Iwama

**Affiliations:** 1 R&D Laboratory, Ezaki Glico Co., Ltd., 4-6-5 Utajima, Nishiyodogawa-ku, Osaka 555-8502, Japan; takashi.mawatari@glico.com (T.M.); yasuo.saito@glico.com (Y.S.); naoki.azuma@glico.com (N.A.); 2 Nihonbashi Cardiology Clinic, Kyodo Bldg. #201, 13-4 Nihonbashi Kodenmacho, Chuo-ku, Tokyo 103-0001, Japan; yiwama@well-sleep.jp

**Keywords:** lactic acid bacteria, *Lactobacillus*, *Lactobacillus helveticus* GCL1815, postbiotics, dendritic cell, humans, symptom, common cold

## Abstract

Background/Objectives: *Lactobacillus helveticus* GCL1815 is a lactic acid bacterium thought to activate dendritic cells. This randomized, placebo-controlled, double-blind study aimed to evaluate the effects of *L. helveticus* GCL1815 on human dendritic cells and the onset of the common cold. Methods: Two hundred participants were divided into two groups and took capsules containing either six billion *L. helveticus* GCL1815 cells or placebo for 8 weeks. Results: In the GCL1815 group, the cumulative incidence days of symptoms such as feverishness, fatigue, tiredness, runny nose, nasal congestion, and phlegm were significantly lower than in the placebo group. Moreover, the change in the expression of HLA-DR on plasmacytoid dendritic cells was significantly higher in the GCL1815 group than in the placebo group at 4 and 8 weeks of intake. The expression of CD86 on plasmacytoid dendritic cells was significantly increased in the GCL1815 group at 4 and 8 weeks compared with before intake. Additionally, the expression of HLA-DR on type 1 conventional dendritic cells was significantly higher in the GCL1815 group than in the placebo group at 8 weeks of intake. The expression of CD86 on type 1 conventional dendritic cells significantly decreased in the placebo group but remained statistically the same in the GCL1815 group after intake compared with before. Conclusions: These results suggest that GCL1815 intake may enhance the response to viruses by activating two types of dendritic cells, thereby preventing the onset of systemic and local common colds in healthy adults.

## 1. Introduction

Infectious diseases are high-risk diseases, with potentially severe outcomes in individuals with a compromised immune system [1]. For example, it is estimated that approximately 3–5 million people worldwide become severely ill with influenza each year [2]. Infectious diseases also lead to substantial economic losses worldwide due to treatment costs and work absences. In the United States, the direct medical cost of treating influenza is reported to be approximately USD 10.4 billion per year [3], and the economic cost of other upper respiratory tract infections is estimated to be USD 40 billion per year, with more than 315 million days of work and school absences per year [4]. For infectious diseases such as influenza, treatment with antiviral drugs is possible [5].

The common cold, one of the most frequent infections, is defined as a mild upper respiratory tract infection that resolves spontaneously. It is reported that children get a cold 7–10 times a year, while adults do so 2–5 times a year [6]. The main cause of the common cold is a viral infection, but more than 200 different viruses are known to cause common colds, including rhinoviruses and adenoviruses [7]. Because common cold symptoms are similar even when caused by different viruses, it is difficult to identify the causative virus based on symptoms alone, and thus it is difficult to tailor the treatment to the virus. Therefore, methods to prevent or reduce the onset of the common cold by modulating the host’s immune function, such as by promoting viral infection defense, are desirable. One useful approach includes the intake of foods and beverages [8] such as green tea, herbs, and mushrooms. Foods containing lactic acid bacteria and bifidobacteria are also among these, and the results of clinical trials have suggested that these bacteria regulate immune function and help prevent the common cold [9,10]. For example, the combined intake of the strains *Lactobacillus gasseri* PA 16/8, *Bifidobacterium longum* SP 07/3, and *B. bifidum* MF 20/5 reduced the duration of common cold symptoms [11], and intake of the *Lacticaseibacillus paracasei* strain Shirota reduced the proportion of medical students with colds before taking exams [12]. In addition to their effect on the common cold, these strains have also been reported to increase CD8^+^ T cells or activate natural killer (NK) cells, which play an important role in suppressing the onset of colds [11,13].

Meanwhile, dendritic cells (DCs), which are antigen-presenting cells found in almost all tissues of the body, also play an important role in suppressing onset of the common cold by regulating both innate immunity, which is the immediate response to invading foreign substances, and adaptive immunity, which is a specific response to foreign substances. DCs are divided into conventional DCs (cDCs) and plasmacytoid DCs (pDCs), which play central roles in the immune response against viral infections. cDCs are further classified into type 1 cDCs (cDC1s) and type 2 cDCs (cDC2s), which induce exceptional activation of CD4^+^ T cells and CD8^+^ T cells through antigen presentation [14]. pDCs are known to produce a large amount of type I interferon (IFN) when they recognize pathogens such as viruses and bacteria [15], directly or indirectly promoting viral replication inhibition as well as CD8^+^ T-cell activation [16].

Lactic acid bacteria are also attracting attention as a means to induce the activation of DCs. For example, *Lacticaseibacillus rhamnosus* GG is reported to activate cDCs and induce IL-12 production *in vitro* [17] and to reduce the number of upper respiratory tract infections in children [18], while the *Lactococcus lactis* strain Plasma is reported to stimulate pDCs via Toll-like receptor 9 [19] and to reduce the cumulative number of influenza and cold-like symptoms in adults [20]. However, not all lactic acid bacteria activate DCs [21], and the immune-modulating functions activated by DCs are known to be strain-specific. Therefore, DCs are considered an important indicator for selecting lactic acid bacteria that can suppress onset of the common cold.

*Lactobacillus helveticus* GCL1815 (hereafter, GCL1815) is thought to activate cDCs [22]. It is reported that cross-presentation and induction of type 1 helper T cell differentiation by cDCs are promoted by type I IFN [16,23] and that cDC function may be reinforced by pDC activation. Because pDCs are also highly responsive to viruses, the present study focused on not only cDCs but also pDCs as key immune indicators in the fight against viral infection. The effects of lactic acid bacteria intake on both human pDCs and cDCs are largely unknown, and the effect of GCL1815, which is expected to have DC-activating capacity, on the onset of the common cold has not been investigated. Therefore, this study investigated the effects of GCL1815 intake on common cold symptoms and immune cells, with the aim of verifying whether GCL1815 can suppress the onset of the common cold in healthy adults.

## 2. Materials and Methods

### 2.1. Participants

Participants were healthy Japanese adults aged 20 to <65 years at the time of consent who fulfilled the inclusion criteria, did not meet the exclusion criteria, and were deemed eligible to participate by the principal investigator. The aim and other details of the study as well as the secondary use of surplus samples were explained to them, and they provided written consent to participate in the study. The inclusion criteria required that participants were fully informed about the purpose and content of the study, had the capacity to provide consent, voluntarily agreed to participate after fully understanding the study’s purpose and content, and provided written consent to take part in the study. The exclusion criteria were (1) history of diabetes, renal or hepatic disease, or other serious illnesses, including thyroid disease, adrenal gland disease, psychiatric disease, autoimmune disease, or other metabolic diseases; (2) inability to abstain from foods containing lactic acid bacteria, bifidobacteria, oligosaccharides, or other bacteria during the study period; (3) regular consumption of foods that can affect immunity; (4) regular alcohol consumption, averaging around 20 g of pure alcohol per day; (5) inability to abstain from alcohol 2 days before each test; (6) presence of food allergies; (7) history of gastrointestinal diseases or gastrointestinal surgery; (8) pregnant, breastfeeding, or planning to become pregnant during the study period; (9) history of drug or alcohol dependence; (10) participation or intent to participate in other studies involving the use of other foods, medicines, or cosmetics; (11) regular use of medicines that may affect immunity or the inability to stop taking them during the study period; (12) hay fever, atopic dermatitis, allergic rhinitis, bronchial asthma, or chronic bronchitis; (13) oral problems involving bleeding or intent to undergo dental or oral treatment; (14) regular engagement in strenuous exercise; (15) intent to travel abroad during the study period; (16) having taken antibiotics within 1 month of the screening test; (17) smoking; (18) having collected or donated more than 400 mL of blood components within 3 months prior to the date of consent; (19) work schedule involving both day and night shifts, working late at night, or fully remote work or blue-collar work; (20) diagnosis of dry mouth; (21) absence of any cold symptoms within the last year; (22) body mass index of <18.5 or >30.0; and (23) determined to be inappropriate as a research participant by the principal investigator.

### 2.2. Test Foods

Capsules containing six billion heat-sterilized GCL1815 cells (test food) and capsules without GCL1815 (control food) were prepared as test food. The inactive ingredients of the test food were starch, calcium stearate, and silicon dioxide and thus the test food did not contain any allergenic substances.

### 2.3. Study Design

The study had a randomized, double-blind, placebo-controlled, parallel-group design. The participants were randomly assigned to two groups (1:1) using computer-generated randomization in blocks of four, with stratification by age, gender, salivary secretory immunoglobulin A (sIgA) levels, expression levels of surface markers on pDCs, and background survey results at screening. The allocation manager assigned participants to either the GCL1815 intake group or the placebo food intake group. The final target sample size was set at 200 based on previous studies reporting reductions in colds with lactic acid bacteria [20,24]. Participants took capsules containing either GCL1815 or placebo once daily for 8 weeks from January 2023 to March 2023. During the study period, both the participants and the observers were blinded to their group allocation. Double-blinding was ensured by labelling the test food with identification numbers only. The primary endpoint was the daily questionnaire on common cold symptoms. Secondary endpoints were expression levels of surface markers on pDCs and cDCs, NK cell cytotoxic activity, and salivary sIgA levels. Other endpoints were the Profile of Mood States 2nd edition (POMS-2) and safety assessment. Blood and saliva samples were collected from the 80 participants who took part in immunological assessments before intake and again at 4 and 8 weeks during the intake period. Surplus blood samples from the immunological assessments were stored at −80 °C and used for additional analysis. Blood samples from the remaining 120 participants were collected before and after the intake period for safety assessment. Additionally, serum cytokine levels were measured for participants with immunological assessments. Samples from participants with immunological assessments whose peripheral blood mononuclear cell (PBMC) counts exceeded the number of cells in the analysis criteria were also measured to determine the expression of surface markers on CD4^+^ T cells, CD8^+^ T cells, B cells, and NK cells. The number of analyzed samples was 40 before intake, 39 at 4 weeks, and 33 at 8 weeks of intake. The study was pre-registered in the University Hospital Medical Information Network Clinical Trials Registry (UMIN-CTR) (UMIN000049616, UMIN000053740) and was conducted at the Nihonbashi Cardiology Clinic (Tokyo, Japan) from January to March 2023. This article adheres to the Consolidated Standards of Reporting Trials (CONSORT) 2010 guidelines.

### 2.4. Questionnaire Analysis

Participants recorded daily symptoms of feverishness, chills, fatigue, low appetite, tiredness, joint discomfort, nasal congestion, runny nose, muscle pain, heavy headedness, throat discomfort, sneezing, cough, and phlegm. Participants rated symptoms as (1) normal, (2) slight, (3) mild, (4) moderate, or (5) severe. Days with mild, moderate, or severe symptoms were counted as symptomatic days, and the cumulative number of days with symptoms was calculated. Additionally, ‘moderate’ and ‘severe’ symptoms were grouped together as serious cases, and ‘mild’ symptoms were taken as mild cases. The numbers of mild and serious symptoms in each of the 14 categories were totaled to assess the proportion of serious symptoms among all symptoms.

The POMS-2 was administered before intake and at 4 and 8 weeks of intake to examine mood states. It consists of 65 questions with responses on a five-point Likert scale, and assess seven states: anger-hostility, confusion-bewilderment, depression-dejection, fatigue-inertia, tension-anxiety, vigor-activity, and friendliness.

### 2.5. Flow Cytometric Analysis

PBMCs were obtained from blood samples using centrifugation. For pDC labelling, PBMCs were stained with anti-CD123 FITC (BioLegend, San Diego, CA, USA), anti-CD304 APC (BioLegend), anti-CD86 PE (eBioscience, Santa Clara, CA, USA), and anti-HLA-DR PerCP-CyTM5.5 (BD Biosciences, Franklin Lakes, NJ, USA). For cDC labelling, CD3^+^ CD14^+^ CD16^+^ cells were eliminated from PBMCs using anti-CD3 (BD Biosciences), anti-CD14 (BioLegend), and anti-CD16 (BioLegend), and the remaining fractions were stained with anti-CD11c FITC (BD Biosciences), anti-CD141 APC (BD Biosciences), anti-CD86 PE (eBioscience), and anti-HLA-DR PE (BD Biosciences). cDC1s and cDC2s were classified based on the presence or absence of CD141 expression. Cells were washed with stain buffer, fixed in paraformaldehyde, and measured using a flow cytometer (BD AccuriTM C6; BD Biosciences). Expression levels of HLA-DR and CD86, indicators of pDC and cDC activation, were measured using mean fluorescence intensity. Sample data with fewer than 50,000 PBMC events were treated as missing values. In the additional analysis, Pacific Blue™ anti-human CD3 antibody (BioLegend), Brilliant Violet 650™ anti-human CD4 antibody (BioLegend), APC/Fire™ 750™ anti-human CD8 antibody (BioLegend), Alexa Fluor^®^ 700 anti-human CD16 antibody (BioLegend), Brilliant Violet 605™ anti-human CD20 antibody (BioLegend), FITC anti-human CD56 (NCAM) antibody (BioLegend), and PE anti-human CD69 antibody (BioLegend) were used to label CD4^+^ T cells, CD8^+^ T cells, B cells, and NK cells by staining. Expression levels of CD69, an indicator of the activation of these cells, were measured using mean fluorescence intensity. Cells were then washed with fluorescence-activated cell sorting buffer and measured using a CytoFLEX S (Beckman Coulter, Brea, CA, USA).

### 2.6. Chromium 51 Release Assay

A chromium 51 (51Cr) release assay was performed to measure the cytotoxic activity of NK cells. K562 cells were labelled with 51Cr (PerkinElmer Japan, Yokohama, Japan), added to PBMCs, and incubated for 4 h at 37 °C and 5% CO_2_. The released 51Cr was measured using a Wizard2 Automatic Gamma Counter (PerkinElmer, Waltham, MA, USA). NK cell cytotoxic activity was expressed as a percentage of cytotoxicity.

### 2.7. Measurement in Saliva

Saliva was collected by having participants hold a Salivet-attached sponge in their mouth for 2 min. The weight of their saliva was measured at the time of collection. The sIgA concentration in saliva was determined using the YK280 Human s-IgA ELISA kit (Yanaihara Institute Inc., Shizuoka, Japan). The sIgA secretion rate was calculated from the saliva weight and sIgA concentration.

### 2.8. Measurement of Cytokine Concentrations

A sandwich enzyme-linked immunosorbent assay was performed to measure the cytokine concentrations in serum supernatants. The aliquoted serum was assayed for cytokines, using the Luminex Discovery Assay (R&D Systems, Minneapolis, MN, USA), S-PLEX Human IFN-α2a (Mesoscale, Rockville, MD, USA), S-PLEX Human IFN-β (Mesoscale), S-PLEX Proinflammatory Panel 1 (Mesoscale), U-PLEX Biomarker Group 1 (Mesoscale), V-PLEX Cytokine Panel 1 Human Kit (Mesoscale), and MESO QuickPlex SQ 120MM (Mesoscale), following the instructions provided with each respective kit. The following parameters were measured: IFN-α, IFN-β, IFN-γ, IL-1, IL-2, IL-4, IL-6, IL-8, IL-10, IL-12, IL-15, IL-17, IL-18, IL-27, a proliferation-inducing ligand, B-cell activating factor, and tumor necrosis factor (TNF)-α1. Samples with values below the detection limit were treated as missing values.

### 2.9. Safety Assessment

The safety of the test food was assessed by testing blood and urine samples obtained from the participants before intake and at 8 weeks of intake. The following parameters were measured: blood pressure, pulse rate, body weight, body mass index, red blood cell count, white blood cell count, hemoglobin, hematocrit, mean corpuscular volume, mean corpuscular hemoglobin volume, mean corpuscular hemoglobin concentration, platelet count, leucogram, total protein, albumin, aspartate aminotransferase, alanine aminotransferase, lactate dehydrogenase, total bilirubin, alkaline phosphatase, gamma-glutamyl transferase, urea nitrogen, creatinine, uric acid, Na, Cl, K, Ca, total cholesterol, low-density lipoprotein cholesterol, high-density lipoprotein cholesterol, triglycerides, glucose, glycated hemoglobin, urine protein qualitative data, urine glucose qualitative data, urine urobilinogen qualitative data, urine bilirubin qualitative data, urine pH, urine specific gravity, urine ketones, and urine occult blood reaction. Qualitative items were quantified as follows: ‘−’ as 0, ‘+−’ as 1, ‘+’ as 2, ‘++’ as 3, and ‘+++’ as 4 for urine protein, urine glucose, urine bilirubin, urine ketones, and urine occult blood reaction. For urine urobilinogen, ‘+−’ was scored as 0, ‘+’ as 1, and ‘++’ as 2. In addition, medical consultations were conducted before intake and at 4 and 8 weeks of intake.

### 2.10. Statistical Analysis

The cumulative incidence days with symptoms based on the daily questionnaire was assessed using the chi-squared test. For immunological parameters, participants’ characteristics and safety parameters, means, standard deviation, and standard errors were calculated using IBM^®^ SPSS^®^ Statistics 27 (IBM Corp., Armonk, NY, USA). Means, standard deviations, and standard errors were presented to the nearest significant figure, with the final digit rounded accordingly. Comparisons between the GCL1815 and placebo groups for each item were assessed with an unpaired *t*-test (two-tailed). Differences between pre-intake and 4 weeks and between pre-intake and 8 weeks of intake were each assessed using a paired *t*-test. For qualitative items, Wilcoxon’s rank sum test was used for comparisons between the GCL1815 and the placebo groups. Missing data were treated as missing values, and no alternative values were used. The level of significance for all analyses was set at 5% (two-tailed test).

## 3. Results

### 3.1. Participant Characteristics

**Figure 1:**
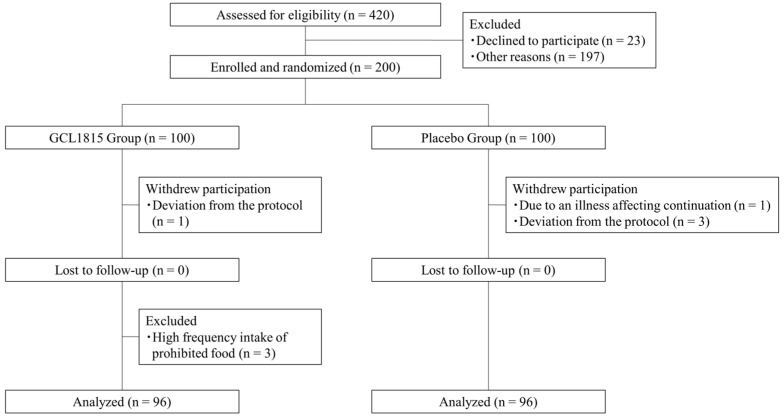
Flow chart of study participation. GCL1815 group, *Lactobacillus helveticus* GCL1815 group.

Of the 420 healthy adults who underwent screening, 200 were deemed eligible, and 100 each were enrolled in the GCL1815 and placebo groups. During the study period, five participants withdrew due to either an illness unrelated to the study, which prevented continuation, or deviation from the protocol. After the study period, three participants were excluded from the efficacy analysis due to high-frequency consumption of prohibited foods, and two of those had undergone immunological assessment. As a result, there were 96 participants in the GCL1815 group and 96 in the placebo group (Figure 1). The full analysis set for safety analysis contained all 200 participants, while the per-protocol set for efficacy analysis contained 192 participants. No differences at baseline were observed in the participants’ characteristics between the GCL1815 group and the placebo group in the efficacy analysis (Table 1).

### 3.2. Common Cold Symptoms and Other Sensory Effects

The effect of GCL1815 on clinical symptoms was evaluated based on the daily questionnaires on common cold symptoms. The cumulative incidence days of common cold-like symptoms was 938 days in the GCL1815 group, which was significantly less than the 1116 days in the placebo group (Table 2). Furthermore, when assessed by symptom, the GCL1815 group showed significantly fewer cumulative incidence days of feverishness, fatigue, tiredness, runny nose, nasal congestion, and phlegm compared with the placebo group (Table 3). The cumulative incidence days of chills was significantly less in the placebo group than in the GCL1815 group. There was no significant difference in the cumulative incidence days of low appetite, joint discomfort, muscle pain, heavy headedness, throat discomfort, sneezing, and cough. The cumulative incidence of mild and serious cases (moderate + severe) was calculated to confirm the palliative effect on severe illness, and the proportion of serious symptoms was assessed. In the GCL1815 group, the proportion of serious symptoms was significantly lower compared with the placebo group (Table 4). These results suggest that GCL1815 intake might have improved common cold symptoms. Furthermore, the results of the POMS-2, which was conducted to assess quality of life, showed a trend toward lower anger-hostility in the GCL1815 group compared with the placebo group at 4 weeks of intake (*p* = 0.088) (Supplementary Material, Figure S1). There were no significant differences between the GCL1815 group and placebo group in other indicators on the POMS-2 at either 4 weeks or 8 weeks of intake.

### 3.3. Effect on Immune Cells and sIgA

**Figure 2:**
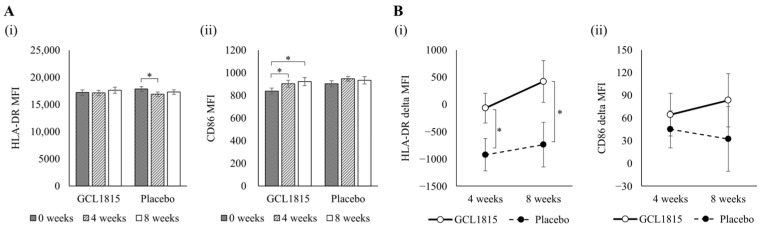
Expression levels of HLA-DR and CD86 on pDCs. Data are means, with the standard errors represented by vertical bars for each group. (**A**) Expression levels of (**i**) HLA-DR and (**ii**) CD86 on pDCs. (**B**) Change in the expression levels of (**i**) HLA-DR and (**ii**) CD86 on pDCs from before intake. The GCL1815 group and the placebo group consisted of 37 and 40 healthy adults, respectively, at 8 weeks. Comparisons of pre- and post-intake within each group were performed using paired *t*-tests, and comparisons between the two groups were performed using unpaired *t*-tests. GCL1815, *Lactobacillus helveticus* GCL1815 group; Placebo, placebo group; MFI, mean fluorescence intensity. * *p* < 0.05.

**Figure 3:**
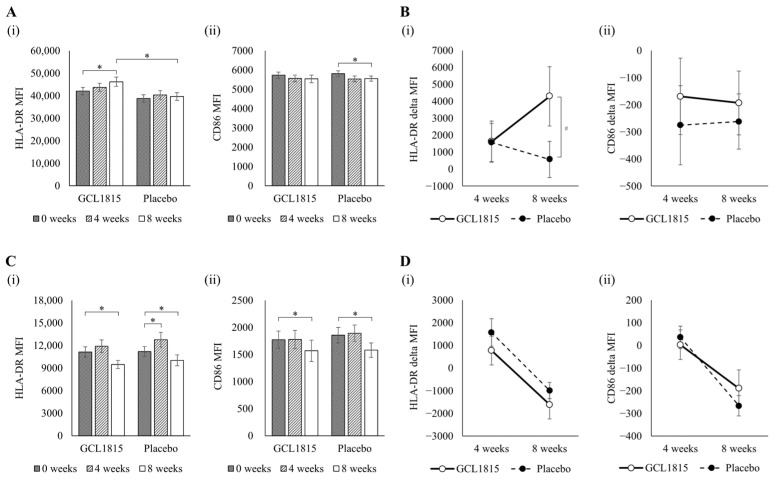
Expression levels of HLA-DR and CD86 on cDC1s and cDC2s. Data are means, with the standard errors represented by vertical bars for each group. (**A**) Expression levels of (**i**) HLA-DR and (**ii**) CD86 on cDC1s. (**B**) Change in the expression levels of (**i**) HLA-DR and (**ii**) CD86 on cDC1s from before intake. (**C**) Expression levels of (**i**) HLA-DR and (**ii**) CD86 on cDC2s. (**D**) Change in the expression levels of (**i**) HLA-DR and (**ii**) CD86 on cDC2s from before intake. The GCL1815 group and the placebo group consisted of 37 and 40 healthy adults, respectively, at 8 weeks. Comparisons of pre- and post-intake within each group were performed using paired *t*-tests, and comparisons between the two groups were performed using unpaired *t*-tests. GCL1815, *Lactobacillus helveticus* GCL1815 group; Placebo, placebo group; MFI, mean fluorescence intensity. * *p* < 0.05, # *p* < 0.1.

Expression levels of surface markers on DCs, NK cell cytotoxic activity, and salivary sIgA levels were measured to assess the effects of GCL1815 intake on immune function. Figure 2A and Figure 3A show the expression of HLA-DR and CD86 on DCs as measured, and Figure 2B and Figure 3B show the change from before intake of the test food. The change in the expression levels of HLA-DR on pDCs from baseline was significantly higher in the GCL1815 group at both 4 and 8 weeks of intake compared with the placebo group (Figure 2B(i)). Meanwhile, the expression levels of HLA-DR on pDCs significantly decreased at 4 weeks of intake in the placebo group but not in the GCL1815 group (Figure 2A(i)). In addition, the expression levels of CD86 on pDCs increased significantly in the GCL1815 group at both 4 and 8 weeks of intake compared with before intake (Figure 2A(ii)). In contrast, the placebo group did not show any significant post-intake changes (Figure 2A(ii)).

The GCL1815 group showed significantly higher levels in the expression of HLA-DR on cDC1s compared with the placebo group, and a significant increase was noted at 8 weeks of intake (Figure 3A(i)). Meanwhile, the change in the expression levels of HLA-DR on cDC1s tended to be higher in the GCL1815 group compared with the placebo group at 8 weeks of intake (*p* = 0.074) (Figure 3B(i)). In addition, the placebo group showed a significant decrease in the expression levels of CD86 on cDC1s after intake, whereas there was no significant decrease in the GCL1815 group (Figure 3A(ii)).

There were no significant differences between the GCL1815 group and placebo group in the expression of either HLA-DR or CD86 on cDC2s (Figure 3C,D). In contrast, both the placebo and GCL1815 groups showed a significant decrease in the expression levels of HLA-DR and CD86 on cDC2s at 8 weeks of intake (Figure 3C). The expression levels of HLA-DR on cDC2s significantly increased at 4 weeks of intake in the placebo group (Figure 3C(i)). However, the changes in the expression levels of both CD86 and HLA-DR on cDC2s were similar in both the GCL1815 group and placebo group (Figure 3D). NK cell cytotoxic activity, salivary sIgA concentration, and salivary sIgA secretion rate did not differ significantly between the GCL1815 group and placebo group at either 4 or 8 weeks of intake (Table 5). Additionally, no significant differences in the expression of CD69 on CD4^+^ T cells, CD8^+^ T cells, NK cells, or B cells were observed between the GCL1815 and placebo groups (Supplementary Material, Table S1).

### 3.4. Effect of GCL1815 Intake on Cytokines

The effect of GCL1815 intake on cytokine concentrations in serum was investigated. The results for representative cytokines related to pDCs and cDC1s [25] are shown in Table 6. The change in IL-12p40 concentration tended to be higher in the GCL1815 group compared with the placebo group at 8 weeks of intake (Table 6). There were no significant differences between the GCL1815 and placebo groups for other items after intake (Table 6, Supplementary Material, Table S1).

### 3.5. Safety Assessment

During the study period, there were 37 adverse events in 23 of the 200 participants, but none was serious. All adverse events were judged to be not causally related to the study food by the principal investigator. Therefore, no adverse effects were reported. After intake, significant fluctuations were observed in albumin and Cl between GCL1815 and placebo groups (Supplementary Material, Table S2). However, these changes were minor physiological variations, and the study food was judged to be safe by the principal investigator.

## 4. Discussion

In this study, the effects of intake of heat-sterilized GCL1815 for 8 weeks on common cold symptoms and DCs in healthy adults were evaluated. Intake of GCL1815 led to the activation of pDCs and cDC1s as well as reduced incidence days of feverishness, fatigue, tiredness, runny nose, nasal congestion, and phlegm. These findings suggest that GCL1815 may promote viral infection responses by activating two types of DCs, thereby suppressing systemic and local common cold incidence in healthy adults.

The common cold is known to cause a wide range of symptoms throughout the body, including runny nose, nasal congestion, sneezing, coughing, fatigue, sore throat, and fever. In addition, symptoms of the common cold change over time, starting with early signs such as fever, fatigue, and sneezing, followed by symptoms such as runny nose, nasal congestion, and cough [7]. As a result, the common cold, with its variety of symptoms and different times of onset, has been reported to cause an average of 8.7 h of lost working time and reduced labor productivity [26]. Given that universal suppression of common cold symptoms is considered important for maintaining worker productivity, 14 of these symptoms were investigated in adults in the present study. GCL1815 showed efficacy in reducing the cumulative incidence days with common cold symptoms (Table 2), particularly runny nose, congestion, and phlegm as local symptoms, as well as feverishness, fatigue, and tiredness (Table 3). The suppressive effect of GCL1815 on the onset of the common colds was not concentrated on specific symptoms but rather extended from early symptoms such as fatigue to late symptoms such as runny nose, suggesting that GCL1815 effectively reduced the number of days with common cold symptoms. It has been reported that *Lacticaseibacillus rhamnosus* CRL1505 has a number of immunomodulatory effects, including activating DCs derived from intestinal Peyer’s patches *in vitro*; increasing the number of CD4^+^IFN-γ^+^ T cells in the small intestine, which subsequently migrate to the respiratory tract to induce local IFN-γ production *in vivo* [27]; and reducing the number of respiratory symptoms in children [28]. In the present study, GCL1815 suppressed a wide range of symptoms, possibly by stimulating the activation of immune cells, starting with DCs in the small intestine, with some of these cells migrating to the upper respiratory tract and inducing the activation of immune cells in local lymph nodes and other sites. Although there were fewer overall incidence days of symptoms with GCL1815 intake (Table 2), there were significantly fewer cumulative incidence days of chills in the placebo group compared with the GCL1815 group. However, this was considered to be an incidental occurrence.

In addition to mild symptoms, GCL1815 might also alleviate more severe symptoms (Table 4). Because more severe infections can lead to complications such as otitis media, sinusitis, bronchiolitis, and pneumonia, the reduction in more severe symptoms is considered important, even for mild infections such as the common cold. In this study, each symptom was rated on a five-point scale, with mild or more severe symptoms being considered symptomatic. Furthermore, among those rated as symptomatic, when serious symptoms (moderate + severe) were analyzed separately from mild symptoms, intake of GCL1815 significantly reduced the proportion of serious symptoms compared with intake of placebo (Table 4). For infants and the elderly, who are at high risk of complications from the common cold, GCL1815 is expected to be useful as a countermeasure against severe colds.

pDCs and cDC1s were thought to play a central role in the suppression of the common cold by GCL1815 (Figure 2 and Figure 3). Considering that common colds are caused primarily by viral infections [7], a DC-driven protective immune response against viruses is important in suppressing the onset of the common cold. pDCs produce large amounts of type I IFN early in the viral response, which promotes apoptosis of virus-infected cells and inhibits viral replication, thereby enhancing resistance to infection [16]. It is also known that pDCs have the ability to present antigens to CD4^+^ T cells and CD8^+^ T cells [29]. It has been recently reported that intake of lactic acid bacteria leads to suppression of colds and pDC activation [24]. Given that GCL1815 also led to pDC activation, it may also induce a high antiviral infection immune response via pDCs. However, because pDCs have been reported to have different reactivity to different viruses as well as a lower antigen-presenting capacity compared with cDCs [29,30], inducing activation of both pDCs and cDCs was considered important as a measure against viral infection. The intake of GCL1815 induced cDC1 activation; however, to our knowledge, the activation of these two types of DCs has not been reported in clinical trials of other lactic acid bacteria. cDC1s induce mainly cytotoxic T lymphocytes and type 1 helper T cell differentiation, whereas cDC2s induce mainly differentiation of type 2 and type 17 helper T cells [31]. In a clinical trial involving the *Lacticaseibacillus paracasei* strain Shirota, there was no activation of cDC1s, whereas significant activation of cDC2s was observed [32]. Although GCL1815 was suggested to induce cDC activation *in vitro* [22], cDC1s and cDC2s were not classified in that study. Thus, it was unclear whether GCL1815 induces activation of one or both. In the present study, GCL1815 was found to specifically induce cDC1 activation, suggesting that GCL1815 enhances the induction of cytotoxic T lymphocytes via antigen presentation to CD8^+^ T cells by cDC1s, ultimately promoting the elimination of virus-infected cells.

The activation of pDCs and cDC1s by GCL1815 was suggested to promote the production of IL-12 (Table 6). cDC1s are well known to induce type 1 helper T cell differentiation and promote the activation of NK cells and macrophages via IL-12. Furthermore, IL-12 is also known to be produced by pDCs [33] and to enhance the cytotoxic activity of CD8^+^ T cells [34]. The enhancement of IL-12 production via cDC1s by lactic acid bacteria has been confirmed *in vivo* using *Lacticaseibacillus rhamnosus* CRL1505. Namely, *L. rhamnosus* CRL1505 induced the expression of CD11c^+^CD103^+^ DCs and CD11c^+^CD11b^high^ DCs, and increased IL-12 production from CD11c^+^CD103^+^ DCs [27,35]. GCL1815 was also found to promote the production of IL-12 by DCs *in vitro* [22]. In the present study, IL-12 levels tended to be higher compared with the control. Thus, IL-12 production might be promoted through GCL1815-induced activation of pDCs and cDC1s, which may promote cytotoxic activity in NK cells and CD8^+^ T cells. In this study, there were no significant differences in the expression of cell surface markers on NK cells or CD8^+^ T cells between the GCL1815 and control, although their expression was higher in the GCL1815 group after intake. For instance, a clinical trial with a larger sample size than this study reported a significant increase in CD8^+^ T cells along with a reduction in cold episodes [11]. Because the sample size in this study was insufficient, there may have been variability between samples, which might be one of the reasons why significant differences in the expression of surface markers on these cells could not be confirmed.

GCL1815-induced activation of pDCs is thought to further enhance the aforementioned cDC1 function. pDCs are important for cDC function. As reported *in vivo*, when pDCs were absent, cDCs did not produce IL-12 [36]. As reported *in vitro*, bacterial stimulation caused pDCs to mature, thereby promoting cDC activation [37]. It was also reported that pDCs might induce cDC1 migration to the site of infection early in viral infection [38]. It is considered that pDCs activated by GCL1815 reinforce the ability of cDC1s to uptake and present viral antigens, and that the combined action of pDCs and cDC1s promote an immune response to viral infection, thereby suppressing the onset of the common cold.

The study had several limitations. Regarding DC activation, the change in the expression of surface markers on pDCs from baseline was significantly higher in the GCL1815 group compared with the placebo group after intake. In contrast, despite considerable variability, the change in the expression of surface markers on cDC1s showed a tendency to be higher in the GCL1815 group compared with the placebo group after intake. Meanwhile, a significant increase in the expression of surface markers on cDC1s was observed after GCL1815 intake compared with before, and the expression of surface markers on cDC1s was significantly higher in the GCL1815 group compared with the placebo group after intake, suggesting that GCL1815 can induce cDC1 activation. In addition, the expression of surface markers on cDC2s decreased in both the placebo group and GCL1815 group after intake compared with before, but the change from baseline was similar between the two groups. Therefore, although the cause of the decrease in the expression of surface markers on cDC2s after intake of GCL1815 was not clear, it was not considered to be the result of GCL1815 intake. Furthermore, symptoms were recorded based on the participants’ self-recognition. Thus, for a more objective assessment of cold prevention, it will be necessary in future studies to conduct tests that include measurements of body temperature and to obtain a medical diagnosis. Further investigation into the effect of GCL1815 in suppressing the onset of the common cold via DC activation is expected.

## 5. Conclusions

This study verified that intake of GCL1815 reduces the incidence of common cold symptoms and activates immune cells. Intake of GCL1815 for 8 weeks significantly induced activation of both pDCs and cDC1s and significantly reduced the cumulative incidence days of common cold symptoms in adults aged 20 to <65 years. These results suggest the possibility that continuous intake of GCL1815 may provide a preventive effect against the onset of the common cold in healthy adults. It is expected that GCL1815 will help to maintain daily physical condition by promoting two types of DC activation, which in turn will improve labor productivity in workers, reduce the severity of colds in the elderly, and reduce school absenteeism in children, thereby benefiting the lives of people of all ages.

## Figures and Tables

**Table 1:** Participant characteristics by group (per-protocol set).

	GCL1815		Placebo		*p*
	Mean	SE	Mean	SE	
Number of participants (Male: Female)	96 (44:52)		96 (42:54)		-
Age (years)	44.8	1.0	44.5	0.9	0.801
Body weight (kg)	61.3	1.0	60.7	0.9	0.646
Body mass index	22.3	0.3	22.2	0.2	0.675
Systolic blood pressure (mmHg)	129.3	1.5	126.0	1.5	0.120
Diastolic blood pressure (mmHg)	84.7	1.4	82.1	1.2	0.154
Pulse rate (bpm)	75.7	1.0	76.9	1.0	0.414

Data represent the baseline means, with the SE shown for each group and the results of unpaired *t*-tests. GCL1815, *Lactobacillus helveticus* GCL1815 group; Placebo, placebo group; SE, standard error.

**Table 2:** Cumulative incidence days with common cold-like symptoms during the study period.

	Symptom (+)	Symptom (−)	*p*
GCL1815	938	4438	<0.001
Placebo	1116	4260	

*Lactobacillus helveticus* GCL1815 group: *n* = 5376 (96 healthy adults × 56 d); placebo group: *n* = 5376 (96 healthy adults × 56 d). Data represent the cumulative incidence days and the *χ*^2^ test results.

**Table 3:** Cumulative incidence days with individual cold-like symptoms during the study period.

		Symptom (+)	Symptom (−)	*p*
Feverishness	GCL1815	17	5359	0.009
	Placebo	36	5340	
Chills	GCL1815	95	5281	<0.001
	Placebo	51	5325	
Fatigue	GCL1815	140	5236	<0.001
	Placebo	267	5109	
Low appetite	GCL1815	25	5351	0.680
	Placebo	28	5348	
Tiredness	GCL1815	312	5064	<0.001
	Placebo	539	4837	
Joint discomfort	GCL1815	72	5304	0.933
	Placebo	73	5303	
Muscle pain	GCL1815	94	5282	0.125
	Placebo	116	5260	
Heavy headedness	GCL1815	163	5213	0.456
	Placebo	150	5226	
Sneezing	GCL1815	211	5165	0.480
	Placebo	197	5179	
Runny nose	GCL1815	360	5016	0.012
	Placebo	428	4948	
Nasal congestion	GCL1815	140	5236	<0.001
	Placebo	253	5123	
Throat discomfort	GCL1815	185	5191	0.190
	Placebo	161	5215	
Cough	GCL1815	117	5259	0.842
	Placebo	114	5262	
Phlegm	GCL1815	62	5314	<0.001
	Placebo	156	5220	

*Lactobacillus helveticus* GCL1815 group: *n* = 5376 (96 healthy adults × 56 d); placebo group: *n* = 5376 (96 volunteers × 56 d). Data represent the cumulative incidence numbers and the *χ*^2^ test results.

**Table 4:** Cumulative incidence numbers of common cold-like symptoms during the study period, categorized by severity.

	Serious (Moderate + Severe)	Mild	*p*
GCL1815	337	1656	<0.001
Placebo	560	2009	

*Lactobacillus helveticus* GCL1815 group: *n* = 1993; and placebo group: *n* = 2569. Data represent the cumulative incidence numbers categorized into two groups, including serious (moderate + severe), and mild, and the *χ*^2^ test results.

**Table 5:** Changes in NK cell cytotoxic activity and salivary sIgA.

		At 4 Weeks			At 8 Weeks		
	Group	Mean	SE	*p*	Mean	SE	*p*
NK cell cytotoxic activity (%)	GCL1815	2.0	2.2	0.427	2.9	1.7	0.664
	Placebo	4.1	1.4		4.0	1.8	
sIgA secretion rate (μg/min)	GCL1815	−3.1	6.4	0.179	−3.2	10.7	0.965
	Placebo	8.3	5.5		−3.7	8.0	
sIgA concentration (μg/mL)	GCL1815	−11.8	9.2	0.060	−9.7	11.9	0.559
	Placebo	11.3	8.0		−1.3	8.3	

Data represent the mean changes from baseline, with the SE shown for each group and the results of unpaired *t*-tests. NK, natural killer; sIgA, secretory immunoglobulin A; SE, standard error.

**Table 6:** Changes in cytokine concentrations.

	Group	At 4 Weeks			At 8 Weeks		
		Mean	SE	*p*	Mean	SE	*p*
IFN-α2A	GCL1815	20.4	3.1	0.155	11.1	4.6	0.809
(fg/mL)	Placebo	11.1	5.7		8.6	9.0	
IFN-β	GCL1815	−46.8	10.5	0.470	−16.5	18.2	0.451
(fg/mL)	Placebo	−56.6	8.4		−33.2	12.4	
IFN-γ	GCL1815	−18.8	188.1	0.596	89.1	330.4	0.840
(fg/mL)	Placebo	−137.1	118.0		195.9	412.2	
IL-6	GCL1815	−182.5	166.5	0.701	−86.3	240.6	0.737
(fg/mL)	Placebo	−97.8	143.3		23.2	217.8	
IL-12p40	GCL1815	−5.1	2.6	0.156	−3.6	3.5	0.080
(pg/mL)	Placebo	−11.4	3.5		−12.7	3.8	
IL-12p70	GCL1815	2.4	18.4	0.139	4.3	46.4	0.538
(fg/mL)	Placebo	38.9	15.9		34.3	14.1	
TNF-α	GCL1815	−51.7	14.5	0.279	−36.0	18.4	0.301
(fg/mL)	Placebo	−77.4	18.5		−64.6	20.3	

Data represent the mean changes from baseline, with the SE shown for each group and the results of unpaired *t*-tests. SE, standard error; IFN, interferon; IL, interleukin; TNF, tumor necrosis factor.

## Data Availability

Datasets generated during the current study and/or analyzed during the current study are available from the responsible author upon reasonable request.

## Supplementary Materials

The following supporting information can be downloaded at: https://www.mdpi.com/article/10.3390/nu17010101/s1, Figure S1: T-scores of Profile of Mood States 2nd edition, Table S1: Changes in expression levels of surface markers on immune cells and cytokine concentrations, Table S2: Safety assessment.
