# Supplementary material for: Lactobacillus helveticus Induces Two Types of Dendritic Cell Activation and Effectively Suppresses Onset of the Common Cold: A Randomized, Double-Blind, Placebo-Controlled Trial
Source: Nutrients. 2024 Dec 30;17(1):101. doi: 10.3390/nu17010101 (PMC11723090; doi:10.3390/nu17010101)
Supplement: Supplementary file 1 [file nutrients-17-00101-s001.zip › Supplementary Material Figure S1.pdf]

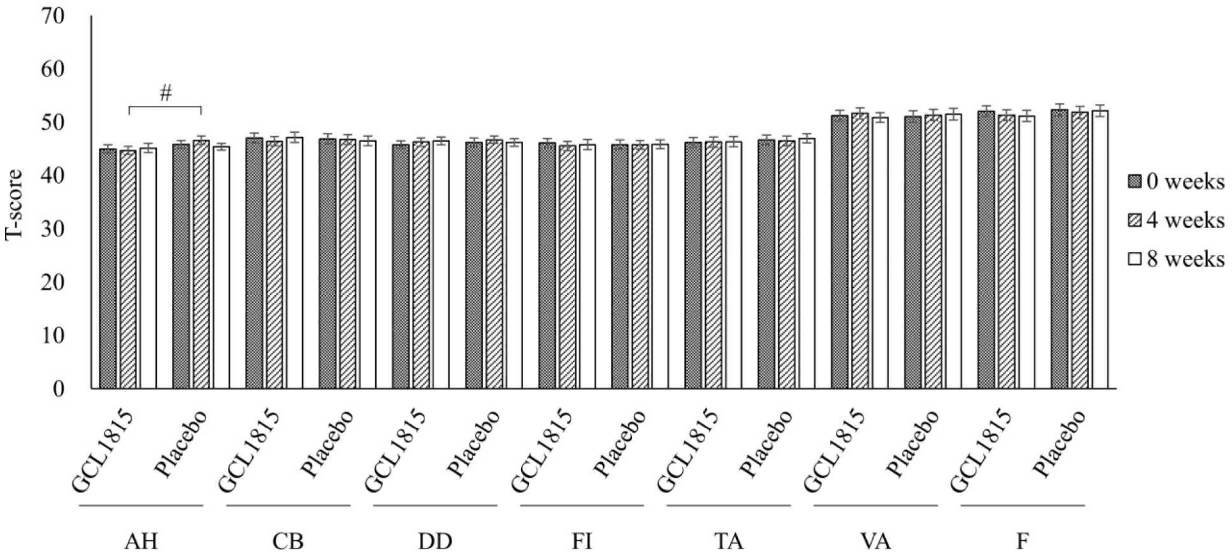

Figure S1. T-scores of POMS-2

Data represent the means, with the standard error shown by vertical bars for each group. Comparisons between the two groups were performed using unpaired *t*-tests. POMS-2, Profile of Mood States 2nd edition; AH, anger-hostility; CB, confusion-bewilderment; DD, depression-dejection; FI, fatigue-inertia; TA, tension-anxiety; VA, vigor-activity; F, friendliness. # $p < 0.1$.
