# Supplementary material for: Lactobacillus helveticus Induces Two Types of Dendritic Cell Activation and Effectively Suppresses Onset of the Common Cold: A Randomized, Double-Blind, Placebo-Controlled Trial
Source: Nutrients. 2024 Dec 30;17(1):101. doi: 10.3390/nu17010101 (PMC11723090; doi:10.3390/nu17010101)
Supplement: Supplementary file 1 [file nutrients-17-00101-s001.zip › Supplementary Material Table S1.pdf]

Table S1. Changes in expression levels of surface markers on immune cells and cytokine concentrations

	Group	At 4 weeks			At 8 weeks		
		Mean	SE	*p*	Mean	SE	*p*
$CD4^+$ T cells	GCL1815	248.2	46.7	0.235	76.5	46.8	0.867
	Placebo	173.0	41.1		62.1	71.2	
$CD8^+$ T cells	GCL1815	378.0	103.8	0.555	70.7	99.7	0.374
	Placebo	468.4	110.4		−127.3	194.6	
NK cells	GCL1815	233.8	113.6	0.445	−201.7	139.2	0.287
	Placebo	81.4	161.1		−497.9	234.7	
B cells	GCL1815	41.1	26.8	0.062	31.9	33.8	0.606
	Placebo	−86.6	60.1		−2.5	56.5	
IL-1β (fg/mL)	GCL1815	4.6	8.8	0.489	34.7	10.2	0.775
	Placebo	18.2	17.4		30.9	8.6	
IL-2 (fg/mL)	GCL1815	−9.3	4.7	0.977	−11.8	5.6	0.823
	Placebo	−9.1	4.9		−13.5	5.4	
IL-4 (fg/mL)	GCL1815	−1.9	4.9	0.948	7.5	4.1	0.479
	Placebo	−1.5	4.5		11.7	4.2	
IL-8 (pg/mL)	GCL1815	−1.5	0.4	0.442	−1.5	0.4	0.442
	Placebo	−2.0	0.6		−2.0	0.6	
IL-10 (fg/mL)	GCL1815	−1.6	53.1	0.634	−82.0	44.7	0.414
	Placebo	−37.1	51.8		−1.8	86.7	
IL-15 (pg/mL)	GCL1815	−0.1	0.0	0.208	−0.2	0.0	0.771
	Placebo	−0.2	0.0		−0.1	0.1	
IL-17 (fg/mL)	GCL1815	−16.5	49.7	0.984	−9.1	44.3	0.621
	Placebo	−17.6	27.6		−38.1	37.9	
IL-18 (pg/mL)	GCL1815	16.5	4.2	0.432	22.2	4.6	0.100
	Placebo	6.6	11.7		−2.2	13.8	
IL-27 (pg/mL)	GCL1815	−51.3	7.0	0.771	44.2	6.8	0.848
	Placebo	−47.8	9.4		42.1	8.7	
APRIL (pg/mL)	GCL1815	161.3	21.0	0.324	173.1	18.9	0.868
	Placebo	130.4	22.9		166.3	35.9	
BAFF (pg/mL)	GCL1815	68.8	10.9	0.242	19.5	13.0	0.536
	Placebo	39.2	22.6		1.5	25.9	

Data represent the mean changes from baseline, with the SE shown for each group and the results of unpaired *t*-tests between the two groups. Data for $CD4^+$ T cells, $CD8^+$ T cells, NK cells, and B cells represent the mean fluorescence intensity of CD69 on these cells. SE, standard error; NK, natural killer; IL, interleukin; APRIL, a proliferation-inducing ligand; BAFF, B-cell activating factor.
