# Supplementary material for: Lactobacillus helveticus Induces Two Types of Dendritic Cell Activation and Effectively Suppresses Onset of the Common Cold: A Randomized, Double-Blind, Placebo-Controlled Trial
Source: Nutrients. 2024 Dec 30;17(1):101. doi: 10.3390/nu17010101 (PMC11723090; doi:10.3390/nu17010101)
Supplement: Supplementary file 1 [file nutrients-17-00101-s001.zip › Supplementary Material Table S2.pdf]

Table S2. Safety assessment

	Group	Baseline			At 8 weeks		
		Mean	SD	*p*	Mean	SD	*p*
Weight	GCL1815	61.4	9.9	0.720	61.2	9.9	0.555
(kg)	Placebo	60.9	8.9		60.5	8.7	
BMI	GCL1815	22.4	2.5	0.616	22.3	2.5	0.483
	Placebo	22.2	2.3		22.1	2.3	
Systolic blood pressure	GCL1815	129.1	14.8	0.152	126.9	14.8	0.641
(mmHg)	Placebo	126.1	14.3		125.9	15.5	
Diastolic blood pressure	GCL1815	84.4	13.5	0.170	81.4	11.5	0.858
(mmHg)	Placebo	81.9	12.0		81.7	13.1	
Pulse rate	GCL1815	75.7	10.2	0.318	75.1	10.8	0.368
(bpm)	Placebo	77.1	9.9		76.5	10.7	
Platelet count	GCL1815	2.7	0.6	0.386	2.7	0.6	0.493
($\times 10^5/\mu L$)	Placebo	2.7	0.6		2.7	0.6	
Red blood cell count	GCL1815	46.2	4.5	0.882	45.7	4.5	0.829
($\times 10^5/\mu L$)	Placebo	46.1	4.1		45.8	4.2	
White blood cell count	GCL1815	53.8	13.4	0.631	52.3	12.2	0.819
($\times 10^2/\mu L$)	Placebo	54.7	15.1		51.9	12.1	
Hemoglobin	GCL1815	13.9	1.4	0.626	13.6	1.5	0.960
(g/dL)	Placebo	13.8	1.5		13.7	1.5	
Hematocrit	GCL1815	43.4	3.8	0.809	42.7	3.8	0.854
(%)	Placebo	43.2	4.0		42.8	4.2	
MCH	GCL1815	30.2	2.1	0.476	29.9	2.1	0.741
(pg)	Placebo	30.0	2.0		29.8	2.0	
MCHC	GCL1815	32.1	1.0	0.378	31.9	1.0	0.710
(%)	Placebo	32.0	1.0		31.9	1.0	
MCV	GCL1815	94.1	5.0	0.750	93.7	5.2	0.783
(fL)	Placebo	93.9	4.8		93.5	4.8	
BASO	GCL1815	0.9	0.4	0.778	0.9	0.4	0.890
(%)	Placebo	1.0	0.4		0.9	0.4	
EOSINO	GCL1815	2.9	2.1	0.795	3.3	2.3	0.902
(%)	Placebo	3.0	2.4		3.4	2.4	
LYMPH	GCL1815	34.8	8.2	0.341	35.4	7.7	0.085
(%)	Placebo	33.7	7.4		33.5	7.5	
MONO	GCL1815	5.5	1.4	0.695	5.5	1.4	0.941
(%)	Placebo	5.6	1.4		5.5	1.2	
NEUT	GCL1815	55.8	9.3	0.470	54.9	9.0	0.124

(%)	Placebo	56.7	7.8		56.7	7.7	
ALP	GCL1815	65.2	17.4	0.521	63.8	18.7	0.782
(U/L)	Placebo	63.7	17.3		63.1	17.4	
ALT	GCL1815	17.7	14.6	0.308	16.1	8.8	0.447
(U/L)	Placebo	16.0	7.8		15.3	6.8	
AST	GCL1815	20.6	6.9	0.431	19.3	5.7	0.099
(U/L)	Placebo	19.9	5.4		18.1	4.6	
LD	GCL1815	171.8	32.5	0.927	173.9	30.0	0.993
(U/L)	Placebo	172.2	24.2		173.9	21.9	
Total bilirubin	GCL1815	0.9	0.4	0.900	0.8	0.3	0.568
(mg/dL)	Placebo	0.9	0.3		0.9	0.4	
γ-GT	GCL1815	22.2	14.8	0.635	20.2	11.9	0.374
(U/L)	Placebo	23.4	20.9		22.4	21.2	
Glucose	GCL1815	85.2	9.4	0.040	84.8	8.4	0.261
(mg/dL)	Placebo	88.0	9.8		86.2	9.0	
HbA1c	GCL1815	5.3	0.3	0.435	5.3	0.3	0.370
(%)	Placebo	5.3	0.3		5.3	0.3	
Total cholesterol	GCL1815	210.3	30.9	0.575	210.6	34.2	0.966
(mg/dL)	Placebo	207.6	37.6		210.4	34.5	
LDL cholesterol	GCL1815	123.2	29.6	0.368	123.2	32.0	0.595
(mg/dL)	Placebo	119.3	32.2		120.8	28.9	
HDL cholesterol	GCL1815	72.1	18.6	0.449	71.9	16.9	0.378
(mg/dL)	Placebo	74.1	19.0		74.1	18.6	
TG	GCL1815	79.7	40.9	0.900	82.4	44.8	0.986
(mg/dL)	Placebo	78.9	53.7		82.5	40.4	
Total protein	GCL1815	7.3	0.4	0.928	7.1	0.4	0.327
(g/dL)	Placebo	7.3	0.4		7.2	0.4	
Albumin	GCL1815	4.4	0.2	0.236	4.4	0.3	0.041
(g/dL)	Placebo	4.5	0.3		4.5	0.2	
Ca	GCL1815	9.4	0.3	0.653	9.4	0.3	0.334
(mg/dL)	Placebo	9.4	0.3		9.4	0.3	
Cl	GCL1815	102.8	2.1	0.063	103.4	1.8	0.013
(mEq/L)	Placebo	103.3	1.9		104.1	1.8	
Creatinine	GCL1815	0.7	0.2	0.801	0.7	0.2	0.972
(mg/dL)	Placebo	0.7	0.2		0.7	0.2	
K	GCL1815	4.2	0.3	0.351	4.2	0.3	0.235
(mEq/L)	Placebo	4.2	0.3		4.2	0.3	
Na	GCL1815	140.7	1.6	0.496	140.4	1.6	0.100

(mEq/L)	Placebo	140.8	1.5		140.7	1.6	
Urea nitrogen	GCL1815	12.7	3.8	0.240	12.6	3.5	0.449
(mg/dL)	Placebo	13.4	4.0		13.0	3.4	
Uric acid	GCL1815	5.0	1.1	0.167	5.1	1.1	0.164
(mg/dL)	Placebo	4.8	1.1		4.8	1.2	
Urine pH	GCL1815	6.2	0.5	0.951	6.2	0.5	0.409
	Placebo	6.2	0.6		6.2	0.6	
Urine specific gravity	GCL1815	1.0	0.0	0.190	1.0	0.0	0.173
	Placebo	1.0	0.0		1.0	0.0	
Urine bilirubin	GCL1815	0.0	0.0	1.000	0.0	0.0	1.000
	Placebo	0.0	0.0		0.0	0.0	
Urine glucose	GCL1815	0.0	0.1	0.570	0.0	0.3	0.325
	Placebo	0.0	0.2		0.0	0.0	
Urine ketones	GCL1815	0.0	0.0	0.156	0.0	0.0	0.150
	Placebo	0.1	0.4		0.0	0.3	
Urine protein	GCL1815	0.1	0.3	0.256	0.1	0.3	0.428
	Placebo	0.2	0.5		0.1	0.3	
Urine urobilinogen	GCL1815	0.0	0.1	1.000	0.0	0.1	0.163
	Placebo	0.0	0.1		0.0	0.0	
Urine occult blood	GCL1815	0.1	0.5	0.543	0.1	0.4	0.207
	Placebo	0.2	0.7		0.2	0.7	

Data represent the mean changes from baseline, with the SD shown for each group and the test results. Comparisons between GCL1815 and placebo groups were performed using unpaired *t*-tests or the Wilcoxon rank-sum tests for qualitative evaluation factors. BMI, body mass index; MCH, mean corpuscular hemoglobin; MCHC, mean corpuscular hemoglobin concentration; MCV, mean corpuscular volume; BASO, basophils in the white blood cell differential; EOSINO, eosinophils in the white blood cell differential; LYMPH, lymphocytes in the white blood cell differential; MONO, monocytes in the white blood cell differential; NEUT, neutrophils in the white blood cell differential; ALP, alkaline phosphatase; ALT, alanine aminotransferase; AST, aspartate aminotransferase; LD, lactate dehydrogenase; γ-GT, gamma-glutamyl transferase; HbA1c, glycated hemoglobin; LDL Cholesterol, low-density lipoprotein cholesterol; HDL Cholesterol, high-density lipoprotein cholesterol; TG, triglycerides; SD, standard deviation.
